# Development of Rubber Packing Element for 105 MPa/215 °C Deep-Well Test Packer

**DOI:** 10.3390/ma15062024

**Published:** 2022-03-09

**Authors:** Yong Chen, Xin Liu, Chen Li

**Affiliations:** School of Mechanical Engineering, Southwest Petroleum University, Chengdu 610500, China; lxswpu@163.com (X.L.); 201921000315@stu.swpu.edu.cn (C.L.)

**Keywords:** packer rubber packing element, numerical simulation, rubber formula, high temperature, high pressure

## Abstract

The rubber packing element is one of the most important parts of deep-well test packers, but the existing rubber packing elements are insufficient to meet the requirements of field use as the stratum temperature and pressure rise as drilling becomes deeper. In this study, a rubber material formulation that meets the actual needs of the field (can withstand a high temperature and high pressure of 215 °C/105 MPa) was designed. Based on this, a mathematical model of the packer’s rubber packing elements was established, and its structure was analyzed using finite element software. Furthermore, the rubber packing elements produced according to this design were verified in an indoor simulation experiment. The results of structural analysis show that the best sealing was achieved when the end-face inclusion angle of the rubber packing element was set at around 40°, the length of the rubber packing element was between 60 and 80 mm, and the hardness was greater than or equal to 90 HA. Under the experimental conditions of 105 MPa and 215 °C, the experimental device stabilized at a pressure for 62 h and the pressure drop was 0.3 MPa, meaning that the elements passed the experiment and, thus, they can go through the setting-down process and function well in normal works. For the rubber packing elements with a sealing capacity and temperature resistance of 105 MPa and 215 °C, respectively, developed in this paper, their sealing reliability was verified through indoor simulation experiments, providing an important guarantee in terms of the smooth implementation of deep-well testing and the completion of operations at high temperature and high pressure.

## 1. Introduction

**Figure 1:**
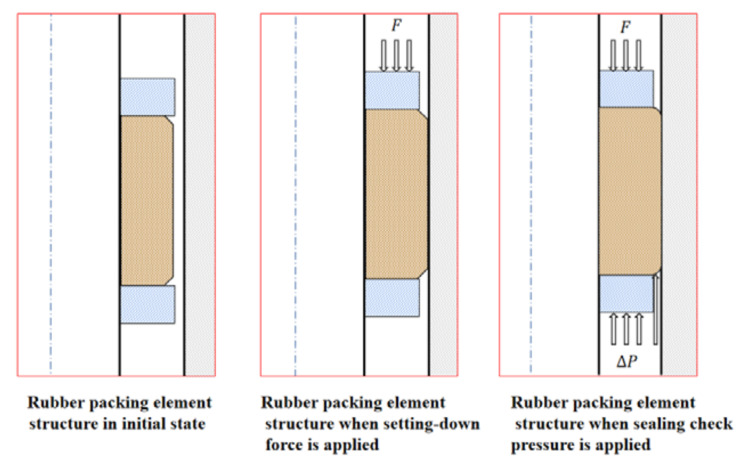
Working principle of the rubber packing element.

The key for packers in terms of providing setting-down sealing lies in their rubber packing elements, and the mechanical properties of rubber packing elements at high temperature are the main factors affecting their sealing ability [1,2]. Figure 1 shows a schematic diagram of the rubber packing elements’ setting developed to realize the sealing function. With the development of the Chinese economy, the demand for oil and gas resources is increasing, and oil and gas exploration and development are increasingly moving towards deep and ultra-deep layers. Many deep and ultra-deep natural gas reservoirs in China have high-temperature and high-pressure conditions. For example, the common characteristics of reservoirs in Tarim Oilfield are as follows: the formation pressure exceeds 105 MPa and the formation temperature exceeds 150 °C. The pressure is 126 MPa [3,4]. At present, the rubber material of rubber products used in oil fields is often strongly eroded under different working conditions (most of them are high temperature and high pressure with a maximum pressure difference above 105 MPa, and the actual temperature may even be above 177 °C), resulting in failure of rubber products and leakage [5]. As the main component of a packer’s rubber packing element is rubber, the sealing performance of rubber packing elements will be greatly affected with the increasing of the ambient operating temperature. If the impact is serious, it will even lead to the failure of the sealing, which will directly lead to the failure of development operations.

In recent years, China has continuously referred to and absorbed foreign leading packer technology, combined with our own geological characteristics, and has developed a variety of new packers and their supporting auxiliary tools. However, at present, the research on the sealing rubber packing elements of packers at home and abroad mainly focuses on the mechanical analysis of sealing rubber packing elements, the design and improvement of their structure, the research on their down-hole working performance [6,7], as well as indoor experimental research on packers [8,9]. Dong [10] studied the parameters of a constitutive model of rubber through a rubber thermal aging test, and based on Yeoh’s model, established a finite element model to analyze the influence of the inclusion angle, temperature (150 °C) and setting load of the packer’s rubber barrel on the sealing performance. Huang [11] discussed the influence of temperature on the constitutive model of hydrogenated nitrile butadiene rubber (HNBR) for packers, and concluded that Yeoh’s model was the most suitable for HNBR through comparative experiments, in addition to obtaining the stress of HNBR at different temperatures (40 °C, 55 °C, 70 °C, 85 °C, 100 °C, 125 °C and 150 °C). Arne [12] tested the tensile behavior of HNBR and FKM elastomers at five different temperatures from −20 to 150 °C, and explored the viscoelastic behavior of elastomers. Xu [13] fitted the constitutive model of hydrogenated nitrile rubber (HNBR) according to the experimental data, and studied the influence of temperature (120 °C) and stress relaxation on the rubber sealing system of the packer using finite element analysis software. Lan [14] used ABAQUS software to numerically optimize the structure of the rubber packing element made of AFLAS material, and verified it by experiments. As a result, a rubber packing element with a high temperature and a high pressure of 170 °C and 70 MPa, respectively, was obtained. Liu [15] established the finite element mechanical model of the packer with material nonlinearity, geometric nonlinearity and double-contact nonlinearity using finite element software, and obtained the contact pressure of rubber and its distribution, deformation, compression stroke and their relationship with axial load, which was verified by the measured test data. Fu [16] systematically studied the effects of the setting load, bottom hole temperature, rubber packing element length, rubber packing element thickness and friction coefficient between the rubber packing element and the casing on the mechanical response of the rubber packing element based on an orthogonal experimental design method. Akulichev [17] proposed a viscoelastic model describing the time–temperature change of permanent deformation caused by compression, which was verified by finite element analysis (FEA) and experimental results. Doughr [18] studied the prediction of the sealing life and reliability of the packers. Alcock [19] studied the mechanical properties of a typical carbon black reinforced hydrogenated nitrile rubber (HNBR) exposed to a mixture of hydrocarbons (toluene, heptane and cyclohexane) at high temperature and high pressure. Gong [20] observed the corrosion behavior of AFLAS, FKM and HNBR rubber in a CO_2_-H_2_S coexistence environment with a high-temperature and high-pressure autoclave, The FKM rubber had the best corrosion resistance and aging resistance. However, these studies and analyses mainly focus on the rubber packing element made of nitrile rubber, and the temperature and pressure involved in the research are all below 100 MPa and 200 °C, which cannot meet the further demand of oil drilling.

In view of the above problems, in this study, a material formula of rubber packing elements that meets the actual needs of the field, and can obtain the physical properties of fluororubber through experiments, was designed. The mathematical model of the rubber packing element made of fluororubber was established, and the finite element method was used to analyze and optimize the structure of the rubber packing elements made of fluororubber designed by ourselves. Finally, the sealing performance of the rubber cylinder was verified by indoor simulation experiments. A set of rubber packing elements of a deep-well test packer at 105 MPa and 215 °C was developed, which provides an important guarantee for the smooth implementation of deep-well test completion operations at high temperature and high pressure.

## 2. Mathematical Model of the Packer’s Packing Elements

### 2.1. Mechanical Analysis of Rubber Packing Elements in Free Deformation Stage

In order to ensure the reliable packing of the packer’s packing element, the minimum axial load required by the packing element should be guaranteed; this is the axial load required to press the packing element sufficiently to just contact the casing wall.

In the stage of free deformation, the deformation of material is a kind of linear deformation and conforms to Hooke’s law; that is, the volume of rubber packing element is unchanged [21], so when the rubber packing element is compressed, the differential equation of radial displacement is as follows:

(1) $$\frac{d^{2}u}{dr^{2}}+\frac{1}{r}\frac{du}{dr}-\frac{u}{r^{2}}=0$$

Let $r=e^{t}$, then t=lnr

(2) $$\frac{du}{dr}=\frac{du}{dt}\frac{dt}{dr}=\frac{du}{dt}\frac{1}{r}=e^{-1}\frac{du}{dt}$$

(3) $$\frac{d^{2}u}{dr^{2}}=\frac{d}{dr}\left(\frac{du}{dr}\right)=e^{-2t}\left[\frac{d^{2}u}{dt^{2}}-\frac{du}{dt}\right]$$

Substituting Equations (1) and (3) into Equation (1) results in:

(4) $$\frac{d^{2}u}{dt}-u=0$$

The general solution of Equation (4) is:

(5) $$u=Ae^{t}+Be^{-t}=Ar+\frac{r}{B}$$

The boundary conditions of Equation (5) are: $\begin{array}{l} u\left|{{}_{r=R}}_{0}\right.=0\\ u\left|{{}_{r=R}}_{{}_{2}}\right.=R_{2}-R_{1} \end{array}$.

Substituting the boundary conditions into (5), the following can be obtained:

(6) $$u=\frac{(R_{2}-R_{1})R_{2}}{R_{2}{}^{2}-R_{0}{}^{2}}r-\frac{(R_{2}-R_{1})R_{2}R_{0}{}^{2}}{R_{2}{}^{2}-R_{0}{}^{2}}\frac{1}{r}$$

where:

u—radial displacement of rubber packing element compression;
$R_{0}$—the inner radius of the packing element;
$R_{1}$—the outer radius of the packing element;
$R_{2}$—the inner diameter of the casing;
r—the distance from any point in the rubber packing element to its axis.

The radial strain of the rubber packing element is:

(7) $$\varepsilon_{r}=\frac{du}{dr}=\frac{(R_{2}-R_{1})R_{2}}{R_{2}{}^{2}-R_{0}{}^{2}}+\frac{(R_{2}-R_{1})R_{2}R_{0}{}^{2}}{R_{2}{}^{2}-R_{0}{}^{2}}\frac{1}{r^{2}}$$

The circumferential strain of the rubber packing element is:

(8) $$\varepsilon_{\theta }=\frac{u}{r}=\frac{(R_{2}-R_{1})R_{2}}{R_{2}{}^{2}-R_{0}{}^{2}}-\frac{(R_{2}-R_{1})R_{2}R_{0}{}^{2}}{R_{2}{}^{2}-R_{0}{}^{2}}\frac{1}{r^{2}}$$

The axial strain of the rubber packing element is: $\varepsilon_{z}$.

Thus, the volumetric strain of the packer’s packing element is: $\theta =\varepsilon_{r}+\varepsilon_{\theta }+\varepsilon_{z}$.

According to the compression characteristics of rubber and the principle that the volume of rubber packing element remains unchanged after deformation, the θ=0; that is,

(9) $$\varepsilon_{z}=-(\varepsilon_{r}+\varepsilon_{\theta })=-\frac{2(R_{2}-R_{1}^{})R_{2}}{R_{2}{}^{2}-R_{0}{}^{2}}$$

Then, the stress in all directions is:

(10) $$\begin{array}{l} \sigma_{r}=\frac{E}{\left(1-2\mu )(1+\mu \right)}\left[\left(1-\mu )\varepsilon_{r}+\mu (\varepsilon_{z}+\varepsilon_{\theta }\right)\right]\\ =\frac{ER_{2}}{1+\mu }(\frac{R_{2}-R_{1}}{R_{1}{}^{1}-R_{0}{}^{2}})(1+\frac{R_{0}{}^{2}}{r^{2}}) \end{array}$$

(11) $$\begin{array}{l} \sigma_{\theta }=\frac{E}{\left(1-2\mu )(1+\mu \right)}\left[\left(1-\mu )\varepsilon_{\theta }+\mu (\varepsilon_{z}+\varepsilon_{r}\right)\right]\\ =\frac{ER_{2}}{1+\mu }(\frac{R_{2}-R_{1}}{R_{1}{}^{1}-R_{0}{}^{2}})(1-\frac{R_{0}{}^{2}}{r^{2}}) \end{array}$$

(12) $$\begin{array}{l} \sigma_{z}=\frac{E}{\left(1-2\mu )(1+\mu \right)}\left[\left(1-\mu )\varepsilon_{z}+\mu (\varepsilon_{r}+\varepsilon_{\theta }\right)\right]\\ =\frac{2ER_{2}}{1+\mu }(\frac{R_{2}-R_{1}}{R_{2}{}^{2}-R_{0}{}^{2}}) \end{array}$$

where:

E—modulus of elasticity;
μ—Poisson’s ratio.

Then, when the rubber packing element is just compressed close to the casing, the compression interface can be integrated:

(13) $$T_{0}=\int_{R0}^{R2}2\pi r\sigma_{z}d_{r}=\frac{\pi ER_{2}}{1+\mu }(R_{2}-R_{1})$$

It can be seen that the minimum setting-down force is proportional to the gap between the sealing element surface and the casing wall before deformation. During initial installation, the greater the gap between the sealing element surface and the casing wall, the greater the minimum required setting-down force.

### 2.2. Mechanical Analysis of Rubber Packing Elements at the Sealing Check Stage

After the rubber packing element is compressed to the casing surface under the action of setting-down force $T_{0}$, an axial load of $T_{1}$ is applied, and the outer side of the rubber packing element and the inner side of the casing form a stable sealing surface from contact to complete contact. This deformation process of the rubber packing element is defined as the constrained deformation stage. According to the research of Dong [22], the contact stress equation between the rubber packing element and casing wall at this stage is:

(14) $$\sigma_{1}\left(z\right)=P_{0}\left\{\tanh\frac{\hat{\lambda }}{2}\sinh\left[\hat{\lambda }\left(\frac{z}{H_{}}-1\right)\right]+\cosh\left[\hat{\lambda }\left(\frac{z}{H_{}}-1\right)\right]\right\}$$

(15) $$P_{0}=\frac{T_{1}}{A}$$

$\hat{\lambda }$—volume deformation coefficient of the packer’s packing element;
A—effective area of the support ring;
H—length of the rubber packing element.

The contact stress on the rubber packing element is generally composed of two parts: one is the uniform compressive stress in all directions, which is equal to the setting-down force on the rubber packing element. The other is the additional stress caused by the axial pressure of the rubber packing element, and the sealing ability of the rubber packing element; that is, the compressive force on the sealing surface depends on this additional force.

After the rubber packing element is stabilized under the setting force of $T_{1}$, it continues to compress the inner and outer walls of the rubber packing element, which is limited by the central tube and casing, and its radial and circumferential strain is zero. According to the research of Jiang [23], the contact stress equation between the rubber packing element and casing wall at this stage is:

(16) $$\sigma_{2}\left(z\right)=\frac{\Delta P}{\cosh\hat{\lambda }}\cosh\left[\hat{\lambda }\left(\frac{z}{H_{1}}-1\right)\right]$$

(17) $$H_{1}=\left(1-\frac{2R_{2}\left(R_{2}-R_{1}\right)}{R_{2}^{2}-R_{0}^{2}}\right)H$$

To achieve sealing, the contact stress must be greater than the pressure difference ΔP; that is:

(18) $$\sigma_{1\min}+\sigma_{2\min}\geq \Delta P$$

(19) $$F_{\;Setting-down\;force}=T_{0}+T_{1}$$

**Figure 2:**
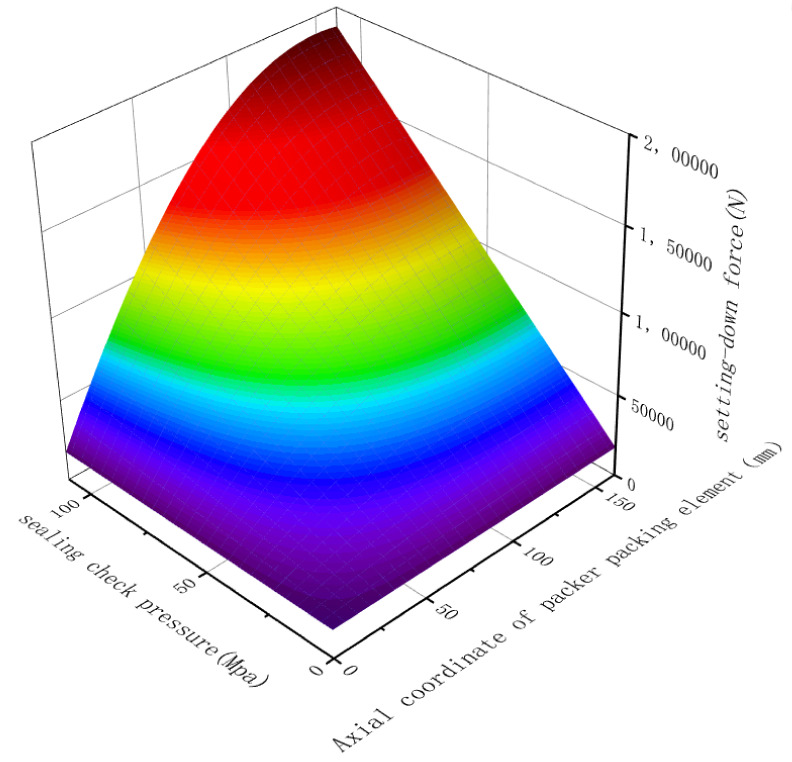
Relational diagram of minimum setting-down force and sealing check pressure.

By combining the formulas (13), (14), (18) and (19) and the structural dimensions shown in Table 1, the three-dimensional diagram of the minimum setting-down force required for rubber packing element sealing with the change of the sealing check pressure as shown in Figure 2 is obtained.

It can be seen from Figure 2 that under the same conditions, with the increasing of the sealing check pressure, the minimum setting-down force required for rubber packing element sealing increases.

## 3. Structural Design of Packer Rubber Packing Elements

**Figure 3:**
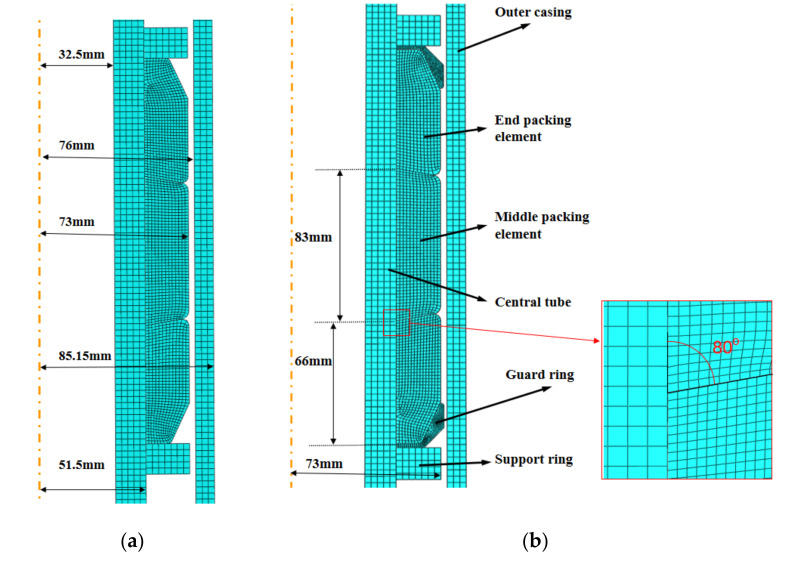
Schematic diagram of rubber packing element model. (**a**). Schematic diagram of rubber packing element without guard ring. (**b**). Schematic diagram of rubber packing element model with guard ring.

The packer rubber packing element is the core component of the packer. In order to ensure the sealing ability of the packer, it is necessary to design the structure of the rubber packing element. The rubber packing element model is shown in Figure 3 below.

### 3.1. Finite Element Model of Packer Rubber Packing Elements

The initial shape of the packer’s rubber packing element has a significant effect on the final sealing effect of the packer. In this study, the effect of the rubber packing element’s structure (transition angle, guard ring and axial length) on the sealing was analyzed using the control variates method, and then the appropriate structure of the rubber packing element for application was designed. A self-developed fluororubber was used as the main material of the rubber packing element, and the geometric and mechanical parameters of the rubber packing element are shown in Table 1.

In the setting process simulation of the packer, material nonlinearity, force nonlinearity and geometric nonlinearity were involved in the calculation due to the characteristics of the rubber material and the contact; therefore, the ABAQUS finite element software was used to carry out the simulation analysis. The following assumptions were made before the simulation analysis: (1) the material behavior of the rubber packing element is elastic; (2) the material behavior of the rubber packing element is isotropic; (3) the rubber packing element will consider the geometric nonlinear effect; (4) the default material of the rubber packing element is incompressible in the calculation.

Among many rubber constitutive models, the Mooney–Rivlin model has been widely used because of its simplicity, few calculation parameters and high analytic efficiency [24]. When constants C01 and C10 of rubber materials are determined, the stress–strain curves obtained with the two-constant Mooney–Rivlin model by simulating different rubber materials using a nonlinear finite element analysis method coincide well with the measured engineering stress curves [25,26]. Therefore, the two-constant Mooney–Rivlin model was adopted in this study, with C10 being 1.0155 and C01 being 0.50778.

The material parameters were assigned to the model based on the Mooney–Rivlin model parameters and the material parameters shown in Table 1. The sliding formulation was defined as finite sliding between the parts, and the friction coefficient between the rubber packing element and the other parts was set to 0.6 (the friction coefficient between the fluororubber and the metal was 0.515 according to the experimental test; considering the complexity of the downhole environment, the friction coefficient was defined as 0.6) and the friction formulation between the metal part was set to “Frictionless” in terms of the tangential behavior; the normal behavior of all the parts was defined as “Hard” contact. The outer surface of the casing, the inner surface of the central tube and the lower Support ring were fixed, and a linearly increasing axial load was applied on the upper support ring to squeeze the rubber packing element downward. The CAX4RH element was adopted for the mesh generation of the rubber packing element, while CAX4H element was used for the mesh generation of the center pipe, the casing, etc.

### 3.2. Effect of End-Guard Ring on Contact Stress of Rubber Packing Elements

The purpose of “shoulder upwarping protection” is to obtain higher contact pressure during the setting down of the packer and to improve the packer’s sealing ability. The so-called “shoulder upwarping protection” means that a guard ring is placed between the rubber packing element and the support ring to prevent and limit the sealing element from “protruding” or “flowing” towards the annular space when the packer is set down, so as to improve and maintain the contact pressure and obtain good sealing performance and reliability [27]. Figure 3 shows an overflow diagram of the packer’s packing element with and without a guard ring under the action of a 60 KN setting-down force.

**Figure 4:**
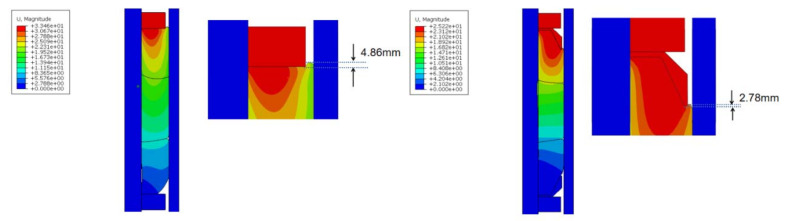
Upwarping of rubber packing element end at 60 KN (**left** picture without guard ring, **right** picture with guard ring).

It can be seen from Figure 4 that when adding a guard ring to the end rubber, the displacement required for the support ring to move down to squeeze the rubber packing element was reduced, In addition, the amount of upwarping of the rubber packing element toward the gap formed between the guard ring and the casing was reduced (from 4.86 mm to 2.78 mm), which was beneficial in terms of the formation of a good sealing surface. It can be seen from Figure 4 that with the addition of a guard ring to the end rubber, the ability of the rubber packing element to flow towards the gap was obviously limited, and it diffused more towards filling the annular space between the rubber packing element and the casing.

From the surface formed by the formula obtained in the previous section, it can be seen that when the rubber packing element was loaded with the setting-down force, the contact stress between the upper end of the rubber packing element and the casing was the largest, and it increased linearly with the increasing of the setting-down force. Therefore, when the guard ring was not installed, under the action of the friction force between the rubber packing element and the casing, the upper area of the rubber packing element tended to stay in the original position when it was squeezed by the support ring, resulting in the failure to fill the annular space between the rubber packing element and the casing. When the guard ring was installed, the displacement required for the support ring to move downward to squeeze the rubber packing element to fill the annular space was reduced, making it possible to avoid the situation in which the rubber packing element could not fill the annular space due to excessive friction.

In order to better compare the sealing ability of the rubber packing element without and with its end-guard ring, the maximum contact stress distribution on the surface of the rubber packing element, the axial compression of the rubber packing element and the overflow under the two working conditions were analyzed by controlling the variable setting-down force.

**Figure 5:**
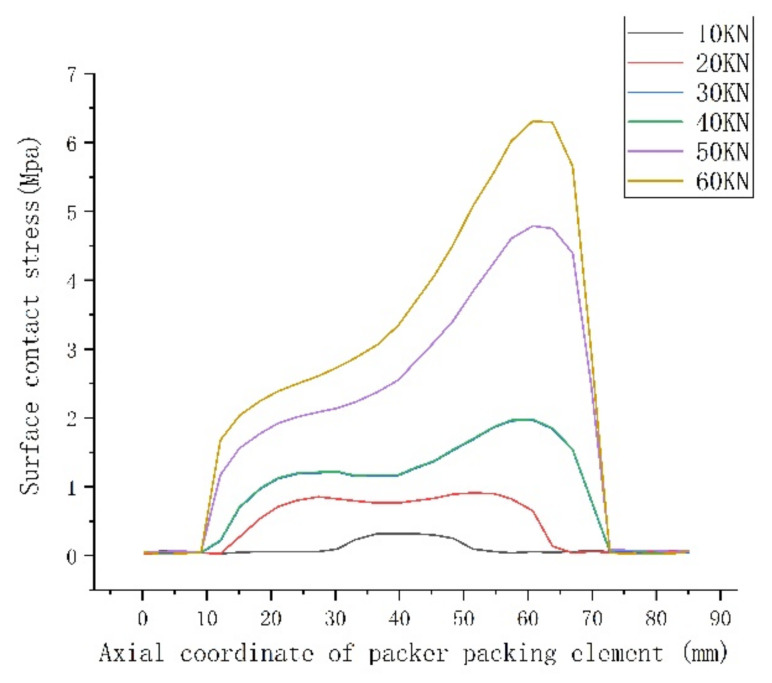
Contact stress of the packer’s packing element without an end-guard ring.

**Figure 6:**
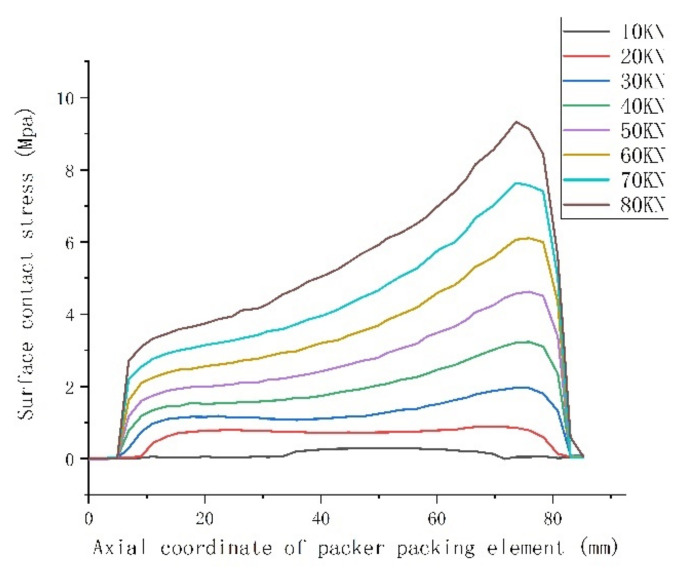
Contact stress of the packer’s packing element with an end-guard ring.

**Figure 7:**
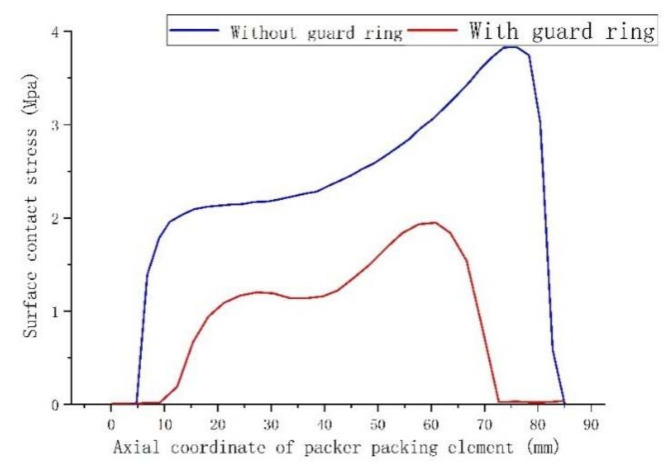
Comparison of contact stress and compression under F = 40 KN setting-down force.

It can be seen from the above Figure 5, Figure 6 and Figure 7 that the maximum contact stress of the packer’s rubber packing element with and without the guard ring was basically the same under the two working conditions, but the contact stress distribution of the rubber packing element with the guard ring was more uniform along the axial direction, and the contact length between the rubber packing element and the casing was longer in the axial direction. To sum up, it can be seen that the rubber packing element with the guard ring at the end could provide better annular sealing.

**Figure 8:**
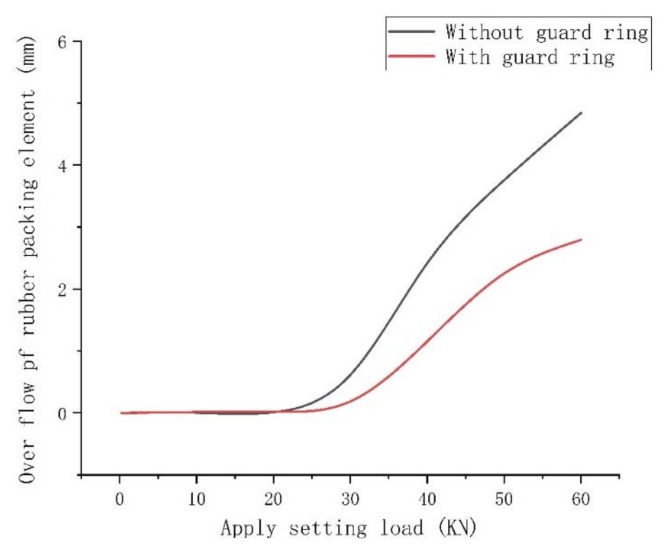
Comparison of rubber overflow with and without a guard ring.

It can be seen from Figure 8 that the packer’s packing elements both with and without the guard ring produced “shoulder upwarping” when the setting-down load was about 20 KN, and this increased as the setting-down load increased, but the overflow amount of the packing element with a guard ring at the end was obviously smaller than that of the packing element without a guard ring. From Figure 5, Figure 6, Figure 7 and Figure 8, the conclusion can be drawn that the end-guard ring can effectively increase the contact area and reduce the “shoulder upwarping”.

### 3.3. Effect of Inclusion Angle of Rubber Packing Element End Face

**Figure 9:**
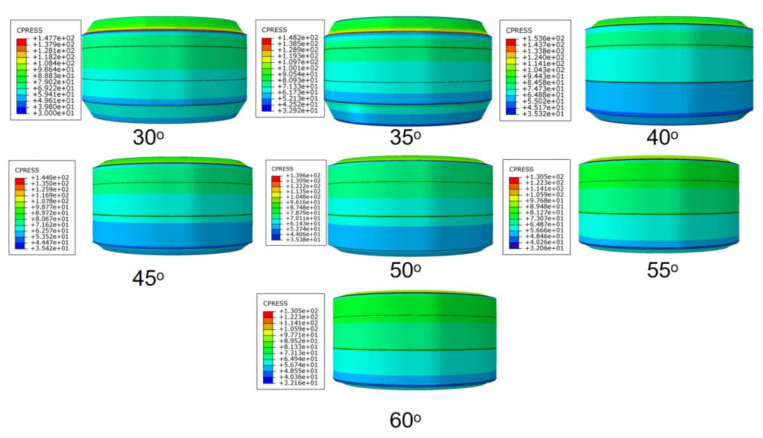
Surface contact stress nephogram of rubber packing element with different inclusion angles under the action of 100 T setting-down force.

**Figure 10:**
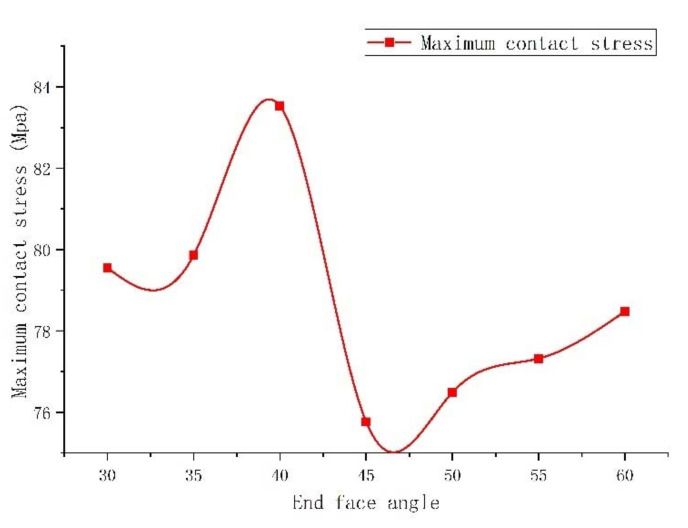
Relationship of maximum contact stress of rubber packing element to inclusion angles.

Rubber is a kind of super-elastomer. Depending on the actual form of the rubber packing element, when the end face of rubber packing element bears a heavy load, the end-face inclusion angle is prone to excessive deformation, specifically the above-mentioned “shoulder upwarping”, which leads to stress concentration at the rubber overflow position, so that the end of the rubber packing element is deformed, cracked or even falls off, and the damaged packing element will reduce the sealing effect [28]. Therefore, the optimization of the end-face inclusion angle plays an important role in increasing the contact stress between the rubber packing element and the casing, and reduces the “shoulder upwarping”. According to the above conclusion, we added a guard ring at the end of the packer’s rubber packing element. Under the axial loading of 100 T setting-down force, the deformation of rubber packing element’s assembly under the extreme pressure of 105 MPa in the annulus (Ø 103–Ø 153 mm) was simulated. Figure 9 and Figure 10 show the contact stress nephogram and the maximum contact stress relationship diagram corresponding to when the inclusion angle of the rubber packing element’s end face changed from 30° to 60°.

It can be seen from Figure 9 and Figure 10 that the maximum contact stress first increased and then decreased with the change of the end-face inclusion angle of the rubber packing element. When the inclusion angle of the end face was 30–40°, the maximum contact stress on the rubber packing element gradually increased and attained its peak when the inclusion angle of the end face reached 40°, and then decreased rapidly. When the inclusion angle of the end face was 55–60°, the maximum contact stress on the rubber packing element increased gradually, but quite slowly.

**Figure 11:**
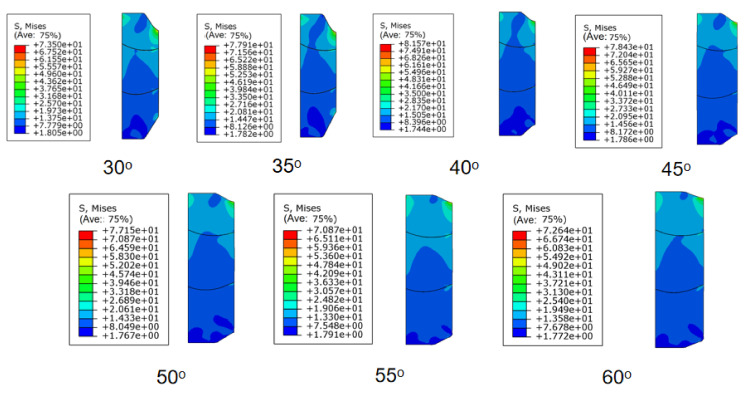
Equivalent stress nephogram of rubber packing element with different inclusion angles under 100 T setting-down force.

As can be seen from Figure 10 and Figure 11, when the rubber packing element was subjected to 100T axial setting-down force, the axial deformation of the rubber packing element’s assembly was uniform, the radial deformation was uniform, the internal groove deformation was uniform, the force on the bonding surface with the casing wall was uniform, and there was no local abnormal stress concentration. The maximum equivalent stress was 81.57 MPa, and the maximum contact pressure of the radial sealing surface was 153.6 MPa.

To sum up, it was established that to ensure the most favorable contact stress, the slope angle should be set around 40°.

### 3.4. Effect of Hardness and Length of Rubber Packing Elements

Because the hardness of a packer’s rubber packing element material is very important for its ultimate pressure when in service, proper hardness can ensure the sealing performance of the rubber packing element when bearing large pressure, and it can ensure that it has uniform deformation, uniform force on the bonding surface with the casing wall and no local abnormal stress concentration when the rubber packing element is compressed [29,30]. In this study, as the contact stress on the surface of the rubber packing element varied as its axial length changed, it was necessary to determine the appropriate axial length by numerical analysis. Packing elements with a 40° end-face angle, a guard ring at the end, a hardness of 60–90 HA and a length of a single piece of between 10 and 95 mm were analyzed with a 100 T load applied at the end of the packing elements.

**Figure 12:**
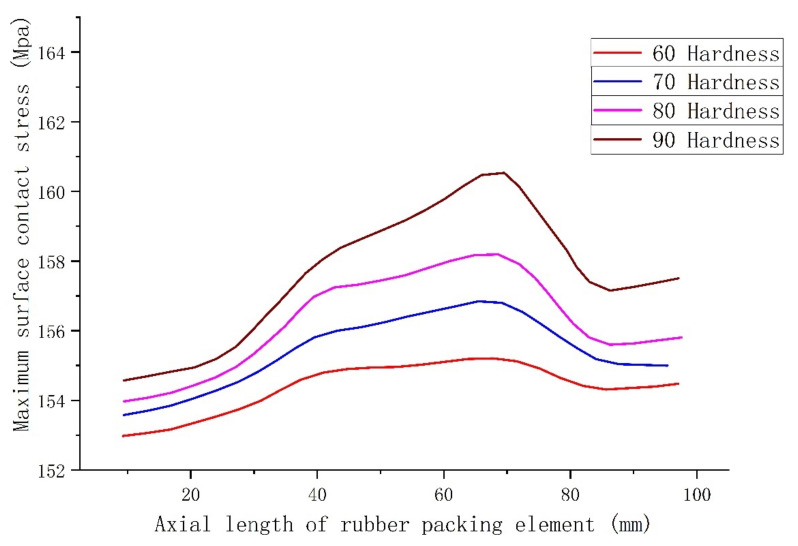
Relationship of length to maximum contact stress of the rubber packing element at different hardness levels.

As shown in Figure 12, the surface contact stress reached its peak value between the lengths of 60 and 80 mm for all hardness levels, and it was found that, in general, the higher the hardness, the better the result. It can be seen that the sealing performance was better when the length of the rubber packing element was 60–80 mm, and again, it was determined that higher hardness led to better results, and hence that a degree of hardness greater than or equal to 90 HA should be selected.

## 4. Formula Design of Rubber Packing Element Material

The rubber material of rubber products used in oil fields is often strongly eroded under severe working conditions (most of them are high-temperature and high-pressure environments with maximum pressure difference above 105 MPa, and a temperature above 177 °C), resulting in failure of rubber products and leakage. Therefore, it is necessary to select materials that are resistant to their service environments as the main components of rubber, and finally to design rubber products that meet application requirements.

### 4.1. Selection of Basic Materials for Rubber Packing Elements

(1) Fluoroelastomer

Carrier rubber is one of the main materials of high-temperature-resistant rubber, and its hardness and strength will affect the mechanical properties of the rubber. Fluorine rubber has excellent corrosion resistance. Generally speaking, it is superior to other rubbers in terms of stability to organic liquids, concentrated acids, high-concentration hydrogen peroxide and other strong oxidants, and has high temperature resistance and oil resistance [31]. Therefore, fluororubber was selected as the carrier rubber here.

(2) Carbon black

Rubber and carbon black can produce physical and chemical adsorption, showing two reinforcing effects: one is to buffer the friction or hysteresis deformation caused by external force; the other is to make the rubber stress distribution uniform. As a result of these two effects, the properties of rubber in all aspects are enhanced; thus, carbon black was selected as the reinforcing agent.

(3) Filler

Rubber fillers mainly include clay, kaolin and calcium powder. In this paper, clay was selected as the filler.

(4) Stabilizer

Adding a small amount of stabilizer can greatly improve the heat resistance of rubber. Inorganic active agents mainly include metal oxides, and MgO is the most important one among them; thus, MgO was selected as a stabilizer [32].

(5) Processing co-agent

Paraffin has good compatibility with fluororubber; it can improve the elasticity of vulcanization and generates no pollution. Therefore, paraffin (paraffin) was used as a processing co-agent.

(6) Vulcanizing agent

Vulcanizing agents can cause rubber molecular chain cross-linking reactions in which linear molecules form a three-dimensional network structure, which reduces plasticity and increases elastic strength. Due to the C-C bond formed by peroxide DADT during vulcanization and cross-linking, the strength increases, which is beneficial in terms of increasing the strength of rubber and leads to better temperature resistance. Thus, DADT was selected as a vulcanizing agent.

(7) Vulcanization accelerator

A vulcanization accelerator is an accelerator that can promote the vulcanization of rubber, shorten the vulcanization time or reduce the vulcanization temperature, reduce the required amount of vulcanizing agent and improve the physical and mechanical properties of rubber. The TAC vulcanization accelerator has the dual functions of vulcanization and promoting vulcanization. Meanwhile, it promotes vulcanization quickly and has strong high-temperature resistance. Therefore, TAC was selected as the vulcanization accelerator [33].

### 4.2. Rubber Packing Element Formula Experiment

Based on the performance parameters of rubber compound under different rubber material ratios, combined with the functions of the upper and lower rubber packing elements, in which the end rubber packing element played a supporting role and the middle rubber packing element played a sealing role, and combined with the strength parameter setting of foreign rubber packing elements and the simulated sealing results, a rubber packing element formula with reasonable comprehensive performance was formed. Table 2 shows the physical performance test results of this formula.

## 5. Indoor Simulation Experiment

As the actual sealing effect of a packer’s rubber packing element cannot be determined by numerical simulation, in order to verify the applicability and reliability of the rubber packing element’s structure obtained by numerical simulation and the rubber formula selected according to the actual working conditions, a high-temperature and high-pressure indoor simulation experiment was carried out on the rubber packing element.

### 5.1. Introduction of Experimental Device and Principle

**Figure 13:**
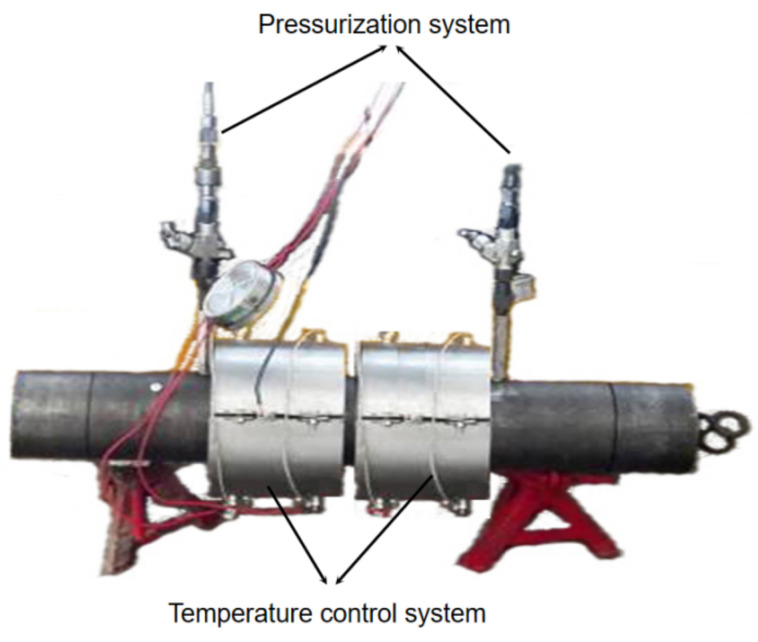
High-temperature and high-pressure experimental device.

**Figure 14:**
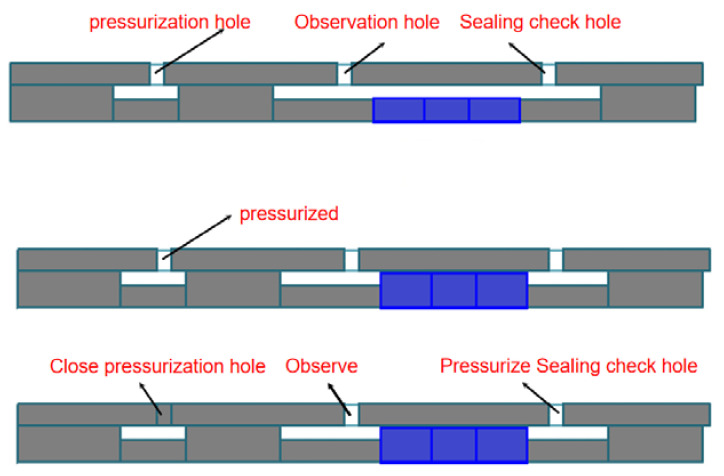
Experimental schematic diagram.

The experimental device and experimental principle are shown in Figure 13 and Figure 14. The experimental device pressurizes through the pressurization hole. After the piston pushes the rubber packing element to set down, the pressurization hole is closed, the sealing check hole is pressurized to verify the sealing, and it is determined whether there is water flowing out from the observation hole to judge the sealing quality. The temperature control system of the experimental device controls the temperature range from 25 °C to 230 °C, the temperature measurement point is inside the device (the observation hole is near the rubber packing element), and the temperature fluctuation is ±5 °C. The pressure control range of the pressurization system is 0~110 MPa, and the pressure change curve can be automatically recorded.

### 5.2. Structure and Physical Parameters of Packer Rubber Packing Elements

**Figure 15:**
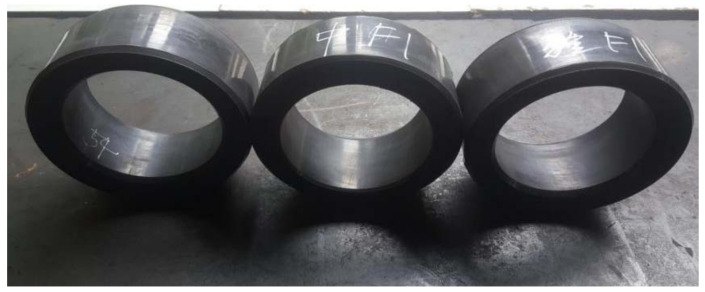
Packer rubber packing elements from the 105 MPa/215 °C experiment.

The rubber packing elements used in the experiment were structured with three rubber packing elements combined together to provide sealing; the inclusion angle of the end rubber packing elements was processed to 40° chamfer and its material was the rubber corresponding to the associated formula, with fluororubber as the main component. The shape of the rubber packing elements was as shown in Figure 15, and the structural size parameters are shown in Table 3.

### 5.3. Experimental Process

Phase One (0–5 h): It was checked whether each component of the test device was installed correctly, whether the pressure and temperature control circuits could operate normally, whether the safety measures were in place and whether the direction of the monitoring image was appropriate.

Phase two (5–7 h): The temperature was raised to 120 °C by the temperature control system and a 20 t setting-down force was applied by injecting water through the pressurization hole.

Phase three (7 h): By injecting water into the sealing check hole to give the initial sealing check pressure of 28 MPa, and continuing to apply heat, the sealing check pressure increased to 62 MPa.

Phase four (7–9 h): When the temperature rose to 185 °C, the pressure was increased to 70 MPa by artificially injecting water from the sealing check hole. Due to the cooling effect of cold water, both the temperature and pressure dropped.

Phase five (9–12 h): Heating was continued. As the temperature rose to 215 °C, the sealing check pressure also rose to 110 MPa, and then the pressure was finally stabilized at about 106 MPa after manual pressure relief.

Phase six (12–74 h): The system voltage stabilization time was 62 h, and there was no pressure drop and leakage during the period.

**Figure 16:**
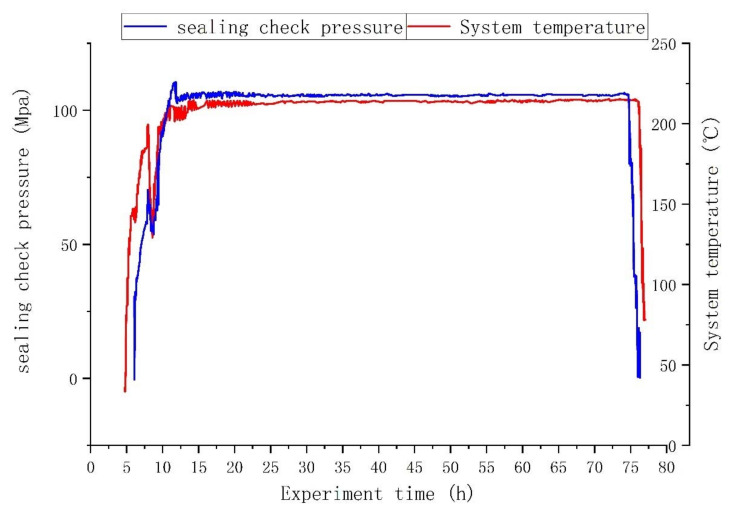
Experimental process curve of the rubber packing element’s sealing.

**Figure 17:**
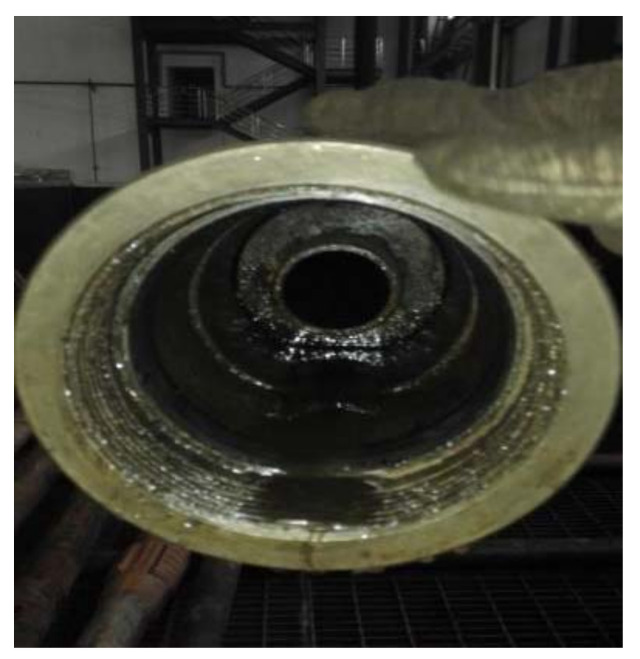
After the experiment, the rubber packing element was still firmly set on the casing.

The relationship between the system temperature and the sealing check pressure and time during the experimental process is shown in Figure 16. Upon completion of the experiment, unsealing was conducted as normal, the packer’s rubber packing element was taken out, and as shown in Figure 17, its appearance was quite good.

### 5.4. Experiment Summary

(1) According to the relational diagram of the minimum setting-down force and sealing check pressure obtained from the calculation results of the mathematical model in Section 2, it can be seen that when the sealing check pressure reached 110 MPa, the required minimum setting-down force was 191,934 N. In order to prevent leakage, during the experiment, the setting-down force was selected as 200,000 N.
(2) In the third section, the equivalent stress distribution of the rubber cylinder was uniform when simulating the actual sealing pressure of 106 MPa, and there was no local abnormal stress concentration.
(3) The experimental results showed that the pressure drop was 0 MPa at an average sealing check pressure of 105 MPa and the system temperature was 215 °C for 62 h. In addition, the elements were still tightly set on the casing after the experiment, without obvious cracking, shedding or damage, showing that the elements passed the experiment and, thus, that they can undergo the setting-down process and will function in normal applications.

To sum up, according to the experimental results, the mathematical model calculation results and the simulation analysis results were consistent, which shows that the mathematical model established in this paper and the results obtained by the ABAQUS software simulation analysis are effective and reliable.

## 6. Conclusions

In this study, the mechanical analysis, structural design and experimental verification of the packer’s rubber packing elements were carried out based on a working environment of 215 °C and a sealing pressure of 105 MPa, with the following conclusions being drawn:

1. It could be determined through mechanical analysis that the minimum setting-down force was proportional to the gap between the surface of the sealing element and the casing wall before deformation, and, in general, the larger the gap between the surface of the sealing element and the casing wall, the greater the minimum required setting-down force. Under the same conditions, the greater the hardness of the material, the greater the axial load required to bear the same pressure difference.
2. It could be determined through numerical simulation that the rubber packing elements with guard rings at the end can provide better annular sealing. The inclusion angle of the end face of the rubber packing element should be set near 40°. The sealing effect is improved when the length of the rubber packing element is between 60 and 80 mm. The hardness should be greater than or equal to 90 HA.
3. A set of high-temperature- and high-pressure-resistant rubber material formulae was designed to meet actual working needs, and the rubber packing elements were made according to the structural analysis results.
4. The sealing ability and temperature resistance of the test packer’s rubber packing elements were 105 MPa/215 °C, and the reliability of the well-bore sealing was verified by indoor simulation experiment, providing an important guarantee in terms of the smooth implementation of the test and the adequate completion of operations in deep wells at high temperature and high pressure.

## Figures and Tables

**Table 1:** Rubber packing element parameters.

	Geometric Parameters			Mechanical Parameters	
	Inner Diameter (mm)	Outside Diameter (mm)	Height (mm)	Modulus of Elasticity (MPa)	Poisson’s Ratio
Central tube	65	103		206,000	0.25
Casing	152	170.3		206,000	0.25
Support ring	103	146	15	206,000	0.25
Middle packing element	103	146	83	18.4	0.49
End packing element	103	146	66	18.4	0.49

**Table 2:** Parameters of the packer’s rubber packing element size for the 105 MPa/215 °C experiment.

Formula Purpose	215 °C/105 MPa End Packer’s Packing Element	215 °C/105 MPa Middle Packer’s Packing Element
Hardness, Shore A	95	86
Tensile strength, MPa	15.15	13.2
Elongation at break,%	130	201
10% modulus, MPa	4.4	2.1
Compression set (15%, 200 °C × 72 h), %	30	27

**Table 3:** Dimensional parameters of the packer’s rubber packing elements in the 105 MPa/215 °C experiment.

Description	Dimensional Parameters	Characteristic
Outside diameter (mm)	146	
Inner diameter (mm)	103	1. Three rubber packing elements were combined together to provide sealing. 2. A spring strengthening structure was added to the end rubber packing elements.
Height (mm)	70 +60 +70(Total length 200 mm)	
Hardness	95° + 86° + 95°	
Strength of rubber packing element material	See Table 2	

## Data Availability

Not applicable.
